# Multi-Component Analysis of Protein- and DNA-Coated Magnetic Nanoparticles Using Electrochemical Impedance Spectroscopy with Interdigitated Electrode Sensors

**DOI:** 10.3390/bioengineering12121334

**Published:** 2025-12-07

**Authors:** Kestley Lutey, Patrick B. White, Hiram Cammon, Miranda Trester, Sue Brumfield, John J. Neumeier, Seth Pincus, Robert W. Haushalter, Joshua Heinemann

**Affiliations:** 1 Montana Microfabrication Facility, Electrical and Computer Engineering Department, Montana State University, Bozeman, MT 59717, USA; kestley.lutey@student.montana.edu (K.L.); mirandatrester@u.boisestate.edu (M.T.); 2 Department of Physics, Montana State University, Bozeman, MT 59717, USA; g79k714@msu.montana.edu (P.B.W.);; 3 Chemistry and Biochemistry Department, Montana State University, Bozeman, MT 59717, USA; sbrumfield@montana.edu (S.B.); seth.pincus@montana.edu (S.P.); 4 Lawrence Berkeley National Laboratory, Berkeley, CA 94720, USA; rwhaushalter@lbl.gov

**Keywords:** cancer, midkine, HIV, electrochemical impedance spectroscopy, nanoparticles, biomarker, bioassay, multiplex assay

## Abstract

The characterization of cancer and other diseases can be aided by the development of reusable electrochemical sensors that provide broad biomarker expression information in real time. We describe an interdigitated electrode (IDE) sensor array that can be used for rapid detection of multiple biomarkers, including human midkine (MDK), HIV gp41 peptide, mAb 7B2, and single-stranded DNA (ssDNA), using electrochemical impedance spectroscopy (EIS) with coated nanoparticles (NPs). These targets represent potential biomarkers for identifying malignant cancer, HIV infection, and DNA mutation. Targets were detected by coating NPs with an antibody, a protein, and ssDNA to capture them from solution. Interacting proteins attached to the nanoparticles were then analyzed with EIS to identify interaction on the surface. In many biological contexts, more than one partner can interact with selected targets, so the determination of the identity of the interacting component is critical for interpretation. In a controlled system, we verify impedance data clusters based on the identity of the protein coated on the surface of the NPs. Data clusters corresponding to protein identity were clearly bifurcated using the impedance spectrum and unsupervised principal component analysis (PCA). NPs clustered based on surface modification, suggesting individual proteins have unique EIS spectral characteristics that can be used for identification.

## 1. Introduction

Biomarker expression data could be enhanced through broad, large-scale quantification of biomarkers for contextually appropriate interpretation. This is critical, as the use of biomarker-based evaluation for disease risk is becoming increasingly used for modern prediction and prevention [1]. There remains a question about how comorbidities affect biomarker presentation and confound diagnostic prediction, as there are a significant number of connections between metabolic and signaling pathways [2]. Broad biomarker data collection with improved hardware could increase predictive accuracy caused by comorbidities like those in HIV-associated cancers. Real-time quantitative data streams of biomarker expression levels and their interaction network could provide dynamic phenotypic maps that can be directed for artificial intelligence (AI)-assisted prediction [3,4]. Detailed phenotypic maps based on biomarker expression would be useful in the identification of different disease subtypes, monitoring therapeutic response, and could improve diagnostic accuracy. To maximize potential, we need technologies capable of rapidly and directly tethering computational predictive analysis to human biomarker responses. This requires new technologies, as existing technologies like mass spectrometry remain broadly inaccessible based on high costs, complexity, and scalability concerns [5,6,7]. One promising technology for the implementation of a multi-biomarker real-time system would be interdigitated electrode (IDE) sensors that utilize electrochemical impedance spectroscopy (EIS). EIS has emerged as a technology for studying protein interaction, which traditionally functions because thin layers of protein bound on gold IDE can be detected through changes in impedance [8,9,10,11,12,13,14]. This technique has broad applicability to multiple molecular levels, as it has been used in many studies to identify and quantify biomarkers through binding interactions with small molecules, proteins/antibodies, and complementary DNA [8,11,12,15,16]. IDEs are fabricated using semiconductor fabrication technology and can be integrated directly into micro-electromechanical systems (MEMS), sharing many of the advantages in scalability and miniaturization. The demonstration of IDE with EIS (IDE-EIS) to detect biomarkers through protein-protein, antibody-protein, or complementary DNA interaction has shown significant detection versatility.

Human immunodeficiency virus (HIV) infection increases the developmental risk of cancers by compromising the immune system [17]. Melanoma cells overexpress the protein biomarkers midkine (MDK) and CD276 (B7-H3), which appear to have increased expression during malignancy [18,19,20,21,22]. MDK modifies the function of several components of the immune system, and recent research shows that blocking MDK can rewire immune cells to resume attack on the tumor [23]. MDK is overexpressed in a variety of cancer types, including melanoma, glioblastoma, neuroblastoma, esophageal, breast, lung, prostate, colon, and others [18,19,23]. In cancer, MDK expression is generally associated with the activation of pro-survival signaling, angiogenesis, and epithelial-to-mesenchymal transition [24]. The occurrence of MDK overexpression does not necessarily indicate cancer, by the large number of interactions it can participate in [24,25]. Additionally, MDK overexpression has a positive role in some contexts, as it naturally helps with wound healing, immune responses [26,27], and has a protective role in HIV infection, confounding simple interpretation [17,28].

Here, we provide evidence using IDE-EIS spectral data as a frequency profile that can identify protein biomarkers and their respective partners when attached to ferromagnetic nanoparticles (FeAgNPs). We report the observation of protein identity-based clustering using EIS data with the unsupervised method of principal component analysis (PCA). Our IDE sensor array could distinguish between multiple protein types bound to magnetic nanoparticles, including antibodies anti-MDK, mAb 7b2, as well as MDK, and HIV receptor peptide gp41 [29]. Additionally, thiolated DNA from Germicidine synthase was attached to FeAgNP to determine if interaction differences could be detected between single-stranded DNA (ssDNA) and complementary ssDNA with a single point mutation, demonstrating broader applicability. Along with multi-target identification, the system has the advantage of fast detection (<3 min) after introduction of the sample and prolonged use with the same sensors performing consistently up to 1400 times over multiple weeks of use. In theory, the IDE-EIS biomarker target flexibility combined with high sensor density could satisfy the requirements needed to provide multi-target real-time biomarker data streams that improve diagnostic accuracy on a single platform.

## 2. Materials and Methods

### 2.1. Materials

Potassium chloride (Fischer Scientific, Pittsburg, PA, USA), AZ1512 photoresist, AZ400T photoresist stripper, AZ 300 MIF developer solution, cysteamine (CYS), N-(3-Dimethylaminopropyl)-N′-ethylcarbodiimide (EDC), Midkine human recombinant, Rabbit anti-Midkine, Bovine Serum Albumin BSA, phosphate-buffered saline (PBS) (NaCl: 136.89 mM, KCl: 2.64 mM, potassium phosphate (dibasic): 10.14 mM, potassium phosphate monobasic: 1.80 mM), 30% hydrogen peroxide, and ammonium hydroxide were obtained from distributors (Merck KGaA, Darmstadt, Germany). 50:1 hydrofluoric acid (JT Baker), gold etchant (Transene Company Inc., Danvers, MA, USA), SU-8 3050 photoresist, and developer were purchased from Kayaku Advanced Materials (Westborough, MA, USA). The photomask was obtained from CAD/ART Services (Artnet Pro Inc., San Jose, CA, USA). The silicone elastomer base and silicone elastomer curing agent (SYLGARD 184) were purchased from Amazon (Seattle, WA, USA). 0.2% heparin solution (Stem Cell Technologies, Vancouver, BC, Canada); 5-thiol disulfides were reduced with immobilized TCEP reducing gel (ThermoFisher, Waltham, MA, USA). Bruker peptide calibration standard II was purchased from Bruker (Bruker Daltonics, Billerica, MA, USA), and 2,5-dihydroxybenzoic acid matrix, potassium ferrocyanide, Tris-Hydrochloride, Ethylenediaminetetraacetic acid (EDTA), magnesium sulfate (MgSO4), and 11-Mercapto-1-undecanol (MCU) were obtained from Sigma-Aldrich (Sigma-Aldrich, St. Louis, MO, USA). Human IgG1 anti-HIV Env mAb 7B2 (anti-gp41 helix-loop-helix region) was created by James Robinson (Tulane University); the antibody used in these studies was a gift from Bart Haynes (Duke University) [29,30]. The peptide representing the target epitope of mAb 7B2 LGIWGCSGKLICTTT was synthesized by Genscript (Piscataway, NJ, USA). Purified DNA oligonucleotides were purchased from Integrated DNA Technologies (IDT Inc., Coralville, IA, USA). The sequences for single-stranded DNA oligonucleotides (ssDNA) were based on the sequence of Germicidine synthase and were as follows:

Oligo 1: (5Thiol_GCS_WT_ FWD):

5′-ThioMC6- ACTCCCGAGACTCCTACACCTCTGGCAACCGCGGTGGCGCAGCAGTGTTCGACATCCTAA-3′

Oligo 2: (GCS_WT_REV):

5′-TTAGGATGTCGAACACTGCTGCGCCACCGCGGTTGCCAGAGGTGTAGGAGTCTCGGGAGT-3′, 100% complementarity, positive control

Oligo 3: (GCS_WT_FWD):

5′-ACTCCCGAGACTCCTACACCTCTGGCAACCGCGGTGGCGCAGCAGTGTTCGACATCCTAA-3′, negative control

Oligo 4: (GCS_R347L_REV):

5′-TTAGGATGTCGAACACTGCTGCGCCACCGAGGTTGCCAGAGGTGTAGGAGTCTCGGGAGT-3′, ~98.3% complementarity single point mutation.

### 2.2. Sensor Array Fabrication

Photolithography photomask design of interdigitated structures and patterning onto devices for IDE sensors were performed using AutoCAD 2021 (Autodesk, San Francisco, CA, USA). These masks were designed with a limited amount of design variations and with as many sensors per fabrication that were practically useful (*n* = 118). Ti/Au-coated substrates were fabricated using physical vapor deposition (PVD) with an AMOD evaporator (Angstrom, Grand Rapids, MI, USA). Substrates were then coated with AZ1512 photoresist using a Laurell spin coater at 4000 rpm for 30 s, followed by a 110 °C soft bake for 1 min, then exposure to light (45 mJ/cm^2^) using an ABM contact aligner. The substrate was then developed in AZ300 MIF developer for 50 s. Once developed, structures were examined using a light microscope to verify photoresist quality before etching. Finally, substrates with photoresist were hard baked at 150 °C for 1 min. IDE sensors were then etched using gold etchant for 40 s, followed by Ti etching with 50:1 hydrofluoric acid for 20 s. Photoresist was then removed using ~100 mL of AZ400T Stripper soak for 45 min at 60 °C.

Polydimethylsiloxane (PDMS) microfluidic gaskets were fabricated using SU-8 3050 on a Si wafer master mold. Briefly, microfluidic master molds were fabricated as follows: Si substrates were cleaned using a PVA Tepla Asher (PVA TePla LLC, Corona, CA, USA) with 600W O_2_/Argon plasma for 1 min. Substrates were then covered with resist using a two-step spin coat program using SU-8 3050, 10 s at 500 rpm, followed by 30 s at 3000 rpm for a final thickness of ~50 μm. Freshly coated substrates were soft baked for 15 min at 95 °C, then exposed to light using a 360 nm long pass filter on an ABM contact aligner (ABM-USA Inc., New York, NY, USA) for 250 mJ/cm^2^. A post-exposure bake was then used on the substrates, first at 65 °C for 1 min, followed by 95 °C for 5 min. Substrates were then developed for 8 min in SU-8 Developer solution, rinsed with IPA, and N_2_ dried. These master molds were then coated with dilute sodium-dodecyl-sulfate in (90%EtOH) to aid in the release of PDMS. PDMS was then mixed from the Sylgard 184 elastomer kit with a 10:1 ratio of base to curing agent in a Flaktek Speedmixer (Flaktek, Landrum, SC, USA) at 3000 rpm for 2.5 min. PDMS was poured over the master mold, degassed, and then cured at 65 °C overnight. Cured PDMS was peeled from the master mold and trimmed. The final microfluidic layer was compression sealed to the IDE sensor surface using an aluminum chassis.

### 2.3. Protein Labeling Directly onto IDE Sensors

As a control, we tested the performance of the IDE sensors with traditional methods using a modified method from Burgos-Flores et al., 2022 [31]. Briefly, IDEs were cleaned using the RCA-1 cleaning protocol for 1 min, then rinsed with deionized water (DI). One hundred microliters of 0.5 M cysteamine were used to cover the IDE sensor surface and left covered at room temperature for 1 h. IDEs were then rinsed with DI water and dried with nitrogen. A solution of 0.5 M EDC solution (pH = 6.5) was reacted with 50 mg/mL protein for two hours. After, 50 mL of EDC-bound protein solution was then used to cover the IDE and was incubated overnight. IDEs were then rinsed with DI and dried with nitrogen. An EIS scan was performed with potassium ferrocyanide as a quality check. IDEs were then blocked using 0.5% (*w*/*v*) BSA and left overnight. Electrodes were then rinsed with DI and dried with nitrogen. For interaction analysis, the complementary proteins/antibodies were compared to a molar equivalent of BSA as a negative control.

### 2.4. Transmission Electron Microscopy of Nanoparticles

Iron- and silver-containing nanoparticles (FeAgNP) were synthesized using a proprietary method (USPTO Application #63/621,258). In microcentrifuge tubes, nanoparticles were prepared for Transmission Electron Microscopy (TEM) by pipetting 10 μL of stock nanoparticle sample (0.635 mg/mL) onto a copper grid and analyzed using an LEO 912 with an accelerating voltage of 100 Kv and EELS (Electron Energy Loss Spectroscopy). ImageJ 1.53 was used to build box and whisker plots of particle size and dispersion, as was previously described by Heinemann et al., 2024 [32].

### 2.5. Magnetic Characterization of Nanoparticles

The following is a summary of the measured magnetic response of FeAgNP, measured with a vibrating sample magnetometer (VSM). The VSM is mounted atop a commercial cryostat, the Physical Property Measurement System (PPMS) from Quantum Design^®^. The sample is glued with Duco Cement^®^ to a sample holder, which in turn is lowered into the sample space containing a superconducting magnet and a coil and connected to a linear transport motor. After lowering the sample into the coil, the magnet is turned on, inducing a magnetization in the sample. The sample is then translated rapidly in the vertical direction by the motor, inducing a voltage in the coil according to Faraday’s Law:

$$v_{coil}=\frac{d\varphi }{dt}=\frac{d\varphi }{dz}\frac{dz}{dt}$$

where *φ* is the magnetic flux through the coil and *z* is the vertical coordinate of the sample. For a sinusoidally oscillating sample position, as is the case with the PPMS VSM,

$$v_{coil}=2\pi fCmAsin(2\pi ft)$$

where *f* is the frequency of the motor, *C* is a coupling constant, *A* is the amplitude of oscillation, and m is the DC magnetic moment induced in the sample by the externally applied magnetic field. The parameter m is the quantity determined in this measurement. A total of three samples were measured: uncoated iron NPs labeled “Fe” and two Ag-coated samples, labeled “Ag1” and “Ag2”. The mass of each sample was determined by weighing each container before and after sample mounting. When not in use, each sample was placed in a glovebox antechamber, which was pumped out and refilled with house N_2_ to reduce oxidation. After each sample was attached to the sample holder and lowered into the pickup coil, the sample was demagnetized by ramping up the external magnetic field and subsequently oscillating the field about zero until only small remnant fields remained (typically 20–50 mOe, an order of magnitude less than Earth’s magnetic field). Measurements of the magnetic moment m were made as a function of the applied field H at temperatures of 300 K (room temperature) and 310 K (human body temperature). Each magnetic moment was normalized to the mass of the sample.

### 2.6. Protein Labeling Nanoparticles

In microcentrifuge tubes, nanoparticles were prepared for protein labeling. They were prepared by the addition of 94.5 μg FeAgNPs to a 150 μL volume of deionized water (pH = 6.5), and after adding CYS to a final concentration of 30 μM, the solution was incubated for 1 h. In a separate tube, the protein to be coated on the FeAgNPs was prepared by the addition of approximately 3 μM protein to a phosphate-buffer solution (pH = 6.5) with a final concentration of 30 μM EDC. The protein-EDC solution was then incubated for 2 h. After incubation, FeAgNPs, now coated with CYS, were magnetically pulled to the bottom of the microcentrifuge tube, supernatant was removed, and then the FeAgNPs were washed with 150 μL DI (pH = 6.5) 3 times and then with PBS (pH 6.5) 3 times. After washing, the EDC protein solution was added to the FeAgNPs and incubated at room temperature overnight. This allowed the EDC-labeled protein to crosslink to the cysteamine coated on the FeAgNP surface.

### 2.7. DNA Labeling Nanoparticles

DNA was attached to FeAgNP using a modified method from [17]. DNA was diluted to approximately 10 μM in ×1 TE-MgSO_4_ buffer (1 μM Tris-HCl, 100 μM, and 0.1 μM EDTA). The 60-mer oligonucleotide DNA probe (Oligo 1) was selected to form a stable duplex with its complementary target (Oligo 2) at room temperature, with minimal interference due to self-complementarity or secondary structure. Oligo 3 was a non-complementary repeat of Oligo 1 and served as a negative control. Oligo 4 contained a single point mutation, R347L, but was otherwise identical to Oligo 2. 5-thiol Germicidine synthetase wt Forward DNA (5-thiol) was immersed in tris-(2-carboxyethyl)-phosphine (TCEP) disulfide reducing gel to break disulfide bonds. 5-thiol was then coated onto FeAgNP by adding 30 μL of the reduced 5-thiol directly onto FeAgNP and mixing thoroughly by pipetting up and down 5 times. The solution was incubated at room temperature overnight. After incubation, FeAgNP, now coated with 5-thiol ssDNA, was centrifuged (1500× *g* for 1 min) to the bottom of the microcentrifuge tube, the supernatant removed, and the FeAgNP was washed 3 times with 50 μL of TE-MgSO4. After washing, 30 μL of Oligo 2, 3, or 4 (~10 μM) was added to the FeAgNP and incubated at room temperature for one hour. Oligo-bound FeAgNP was then spun down in the centrifuge (1500× *g* for 1 min), and the wash cycle was repeated to remove any unbound oligo before resuspending the FeAgNP in 150 μL of TE-MgSO_4_ before analysis.

### 2.8. Principal Component Analysis

Impedance values were isolated and scaled before undergoing principal component analysis. A program script written in Python 3.14.1 extracted real and imaginary impedance data for all EIS tests uniformly across all sensors (Supplementary Figure S1). Every sample analyzed resulted in a column of 20 impedance values at their respective frequency, which were scaled between −1 and 1. This enabled data from different sensors to be compared despite sensor-to-sensor variations that might occur. The data was then transformed into three principal components using the Scikit-learn library and plotted with Matplotlib 3.10.8 [33,34,35]. Multivariable analysis using PC1, PC2, and PC3 values for each sample was simultaneously analyzed under Statsmodels MANOVA in Python [36]. Analysis provided F-statistics and *p*-values for each Wilks’ Lambda, Pillai’s trace, Hotelling–Lawley trace, and Roy’s Greatest root evaluation; data are available in Supplementary information. Variance ratios between the principal components were determined with SciPy.Stats [37].

### 2.9. System Architecture

**Figure 1:**
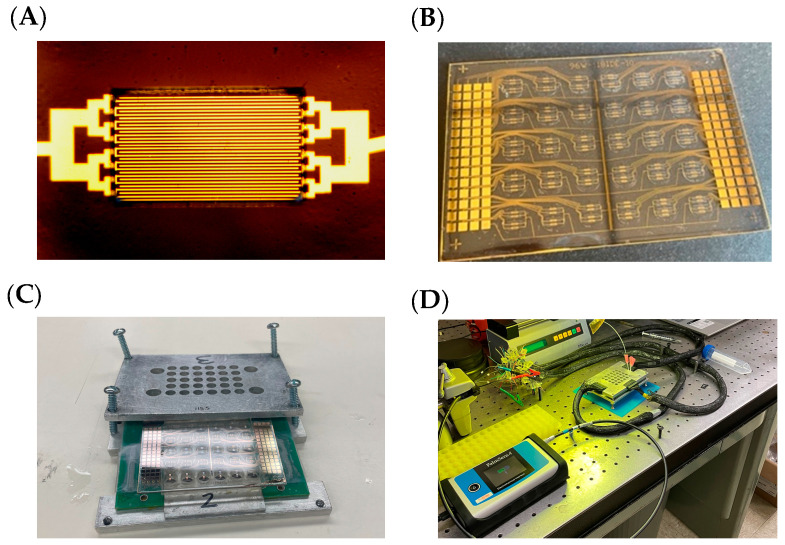
IDE system architecture. (**A**) Microscope image of an individual IDE sensor with 32 (16 × 2) gold interdigitated fingers. (**B**) Photo of 118 sensors laid out in an array for interfacing to (**C**) a 30-well format chassis. (**D**) Photo of overall system setup.

Figure 1C,D show a general representation of the EIS system. The system consists of a Palmsense impedance analyzer (Palmsense BV, Houten, The Netherlands) with an MUX-8 R2 8-channel multiplexor, which can be used to assess 8 sensors together sequentially. The sensors were connected to the Palmsense using a chassis consisting of a printed circuit board (PCB) with gold pogo pins whose layout was originally designed for the DropBot [38]. The chassis stack consisted of two custom-machined aluminum plates holding together the IDE sensor array with a PDMS gasket containing wells into which the samples can be pipetted. The PDMS gasket was pressed tightly against the IDE array by adding tension to the machine screws located around the periphery of the chassis. This created a compression seal between the sensor array and PDMS gasket, preventing solutions from leaking out of the wells with the sensors located at the bottom.

### 2.10. System Calibration and Impedance Measurements

Calibration of each unlabeled sensor was performed with phosphate-buffered saline (PBS) at a pH = 7.5. The EIS was run with 0 VDC bias with 75 mV ADC field between 1 and 1000 Hz in most cases. The potential used was higher than in other traditional studies using EIS. We found that we had better reproducibility with the higher 75 mV potential than with lower values, such as 50 mV, which was also tested. The optimal potential and frequencies were selected based on responsiveness and reproducibility. Because of limitations in the fabrication process, an 80% yield for sensor fabrication was observed, with approximately 20% of sensors disqualified for testing purposes because of inconsistencies or errors. Sensors were exposed to samples in a very controlled sequence to ensure reproducible performance, and proper washing of sensors between samples was undertaken with deionized water (DI). The Palmsense EIS analyzer MUX-8 multiplexor (Palmsense BV, Houten, The Netherlands) allowed 8 sensors to be connected simultaneously (Figure 1D). The purpose of the design of four sensors per well allowed each well to either contain an interacting protein pair or a negative control (proteins that do not interact) that could be tested side-by-side for comparison. The multiplexing array was used to obtain reliable data from each sample condition in quadruplicate simultaneously. Additionally, for each sensor in every sample condition, a triplicate measurement was performed, and every sensor had an EIS profile made for every experimental condition to ensure consistency between sensors. Sensors were washed 3x with DI water by pipetting up and down 5x to help wash the sensors between samples.

### 2.11. Mass Spectrometry

The mass spectrometry analysis used an untargeted approach where molecular features (*m*/*z*) from ionization of standards were used to verify successful synthesis and interaction. Protein-coated nanoparticles were directly analyzed using Matrix Assisted Laser Desorption Ionization (MALDI) on a Bruker Autoflex (Bruker, Billerica, MA, USA). Matrix controls, protein standards (BSA, MDK, anti-MDK, mAb-7B2, and gp41), and protein-coated nanoparticles were analyzed. The samples were dried onto the MALDI plate and coated with 2,5-Dihydroxybenzoic acid (DHB), dissolved to saturation in 50% acetonitrile. An amount of 0.5 μL of the DHB solution was added to every protein spot on the MALDI plate and allowed to dry. As a final step, 0.5 μL of Bruker peptide calibration standard II was added to each spot as an internal standard. Sample spots were put onto pads and ionized using around 60% laser power. The mass range was from 100 to 3200 *m*/*z* in most cases. Using known amounts of protein standards, unique molecular features (*m*/*z*) correlated to each protein identity were extracted using Bruker FlexAnalysis 3.3.80.0 software’s mass list function to create a “mass fingerprint,” specific to each protein (BSA, MDK, anti-MDK, mAb-7B2, and gp41). The mass list was curated by removing any redundant masses that were shared between samples to subtract non-specific or background ions. A list of unique masses correlated to each protein was used to verify the presence or absence of protein in each step of synthesis or interaction. A table of molecular features (*m*/*z*) was correlated with each protein or sample type and was used to verify the presence or absence of protein targets (Table 1).

### 2.12. Zeta-Potential Measurements

Zeta-potential measurements were performed on a Zeta-Meter 4.0 (Zeta-Meter Inc., Harrisonburg, VA, USA). A standard solution of Min-U-Sil was used to ensure the instrument operated in the expected range. Three different solutions containing FeAgNP nanoparticles were analyzed to assess changes due to surface coating: bare nanoparticle control (NP-C), nanoparticles coated with cysteamine (NP-CYS), and nanoparticles coated with cysteamine and reacted with EDC-labeled BSA (NP-BSA). Nanoparticles were diluted in 35 mL of DI water to a concentration of 0.35 mg/mL. A volume of 25 mL was filled into the sample holder and placed under the microscope. The instrument scale was set, and power was set to 100 mV, and a controller was used to time the particle drift (*n* = 10) for each sample.

## 3. Results

### 3.1. Device Fabrication

The goal of the research was to fabricate a sensor array with the ability to run biological experiments with multiple replicates to observe the potential and limitations of EIS for the determination of biomarker presence through interaction detection. The overall design of the sensors used a 7 mm diameter well, with each well containing 4 IDE sensors for a total of 30 wells and 118 individual IDEs available per fabrication (two channels were designated for bussed counter electrodes). The layout of the sensors is based on the DropBot system pogo pin connector originally designed for a digital microfluidic platform [38].

The connector consists of a PCB with gold spring-loaded pogo pins for making contact between the connector and our fabricated IDE array (Figure 1B). The IDE sensor arrays were fabricated on a 2 × 3 in. glass microscope slide by depositing a 20 nm Ti adhesion layer and 100 nm gold thin film. The thin films were then patterned into the IDE array using photolithography and wet etching. The individual sensors consisted of a total of 32 interdigitated fingers, with 16 fingers connected to the working electrode (WE) and 16 fingers connected to the counter electrode (CE). The width of the fingers was 5 μm with a length of 833 μm, with a 5 μm gap between the fingers (Figure 1A). The sensors were arranged into 30 wells, with four individual sensors per well for a total of 120 sensors. The DropBot has 120 pogo pin connectors, which would typically mean only a maximum of 60 sensors, as each WE and CE would require a unique channel. To increase the sensor density, we instead bussed all the CE channels into two redundant channels/pads. This allowed us to have 118 total functional sensors with two pogo pin connectors dedicated as a bussed CE. The chassis, which clamped the IDE sensor array to the DropBot connector, consisted of two machined plates of aluminum that were used to press a 0.5 cm thick PDMS gasket with wells punched through to the sensors. The chassis is held together by 4 machine screws (10–24 thread, 1 in. length) on each of the four corners. The chassis is connected to the Palmsense for EIS using the DropBot PCB layer wire connectors. The wire connectors are stripped on one side to allow the wires to be interfaced to the Palmsense multiplexor alligator clips. The multiplexor allows 8 sensors to be connected simultaneously, allowing four sensors from two different wells to be connected simultaneously or in alternate configurations (e.g., two sensors in four different wells).

### 3.2. Labeling and Testing IDE Sensors

**Figure 2:**
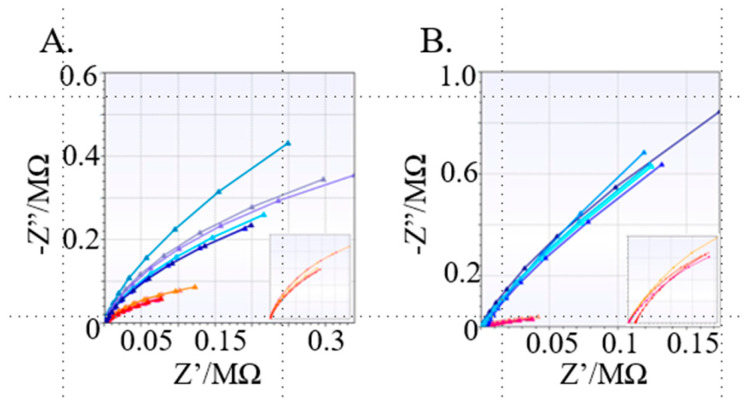
Nyquist plots of IDE sensors coated with interacting proteins (blue) or BSA controls (red), insets: contain closeup of the BSA controls. (**A**) Nyquist plots sensors coated with mAb 7B2 exposed to BSA (red) or gp41 antigen (blue). (**B**) Nyquist plots of sensors coated with gp41 antigen exposed to BSA (red) or mAb 7B2 (blue).

The original strategies for evaluating protein interaction on the sensor surface involved coating the sensors with an antibody or epitope. The advantage of the IDE array format with 4 sensors in each well allowed us to test the robustness and reproducibility of the results with multiple sensors easily using the multiplexor. The goal of the experimentation was to determine if interaction can be detected on the IDE array, like protein-coated single IDE sensors reported in previous studies [31]. The IDE array has some variable factors that need to be accounted for that are not observed in single-sensor setups. The IDE array is not completely uniform, as each sensor has a unique electrical path combined with minor variations in fabrication. The use of 2 × 3 in. glass microscope slides allowed for three slides to be loaded onto the substrate chuck simultaneously using Kapton tape. This significantly reduced the time and cost. In the final fabrication process, the IDE arrays resulted in about an 80% yield of successful sensors, where some variation typically occurred during the chemical etching step. This resulted in some variability in finger width, designed to be 5 μm but having a variation of +/−10% in most cases. Sensors sometimes failed due to fabrication errors but were easy to identify, as typically there was no contact or there was a region between the fingers that did not etch completely. The incomplete etching closes the circuit between the WE and CE and was easily identified because the sensors would not be impeded. These nonfunctional sensors could be identified using just a drop of DI water over the sensor and touching the contact pads of the sensor with a Fluke 177 multimeter (other multimeters were not reliable) to measure direct current (DC) resistance between the WE and CE. A value of approximately 2–3 MΩ DC was ideal for functional sensors when exposed to DI. Variability of impedance profiles due to subtle fabrication differences was typically observed when comparing the impedance response of sensors to the same samples (Figure 2). Despite these shortcomings, consistent trends were easily identified as the change in impedance was typically significantly above sensor-to-sensor variability. Figure 2 shows Nyquist plots of multiple sensors exposed to the same proteins, BSA, versus interacting proteins, mAb 7B2, and gp41 antigen. The Nyquist plots clearly show trends that are distinct for each interacting protein versus equivalent amounts of BSA (negative control). The labeling protocol used was modified from [31]. This strategy used a CYS/EDC approach, where CYS was used to coat the surface of the gold electrode (CYS-IDE), while simultaneously a stoichiometric ratio of EDC to protein (P) was used to label amine groups on the protein (EDC-P). After a brief incubation period, the CYS-IDE is rinsed, and EDC-P is added, crosslinking the protein to the surface of the IDE. The sensors were then rinsed with PBS (pH = 7.5) and exposed to a 60 μL BSA control, gp41 antigen, or mAb-7B2 at a 3 nM concentration. Interaction partners mAb-7B2/gp41 antigen were scanned using EIS in either configuration (Figure 2). IDE sensors were coated with one interaction partner and exposed to a BSA control (red) or interaction partner (blue), and impedance was recorded as Nyquist plots. A clear distinction can be made between the sensors exposed to the interacting partner as a positive control (each blue color line corresponds to a unique IDE sensor) and sensors exposed to BSA negative controls (each red color line corresponds to a unique IDE sensor) in each experiment. The results from these sensors provide evidence that the IDE array can detect interaction-based changes when compared to nonspecific BSA controls.

### 3.3. Characterization of FeAgNP and Testing with IDE Sensors

We observed significant limitations in the traditional IDE setup for detecting protein interaction, to include: (1) protein-coated IDE sensors could only be used to detect a single interaction accurately; (2) the protein-coated IDE sensors need a significant amount of preparation and incubation time during testing; and (3) the testing setup was not ideal for interfacing directly to future in vivo systems. These limitations reduce the usefulness and full potential of protein-coated IDE for interaction detection in cases like biomarker detection. Because of these limitations, we decided to try a new strategy using uncoated IDE sensors to analyze protein-coated FeAgNP magnetic nanoparticles. Instead of coating the sensor surface with protein, we coated FeAgNP nanoparticles, then used the protein-coated FeAgNP to capture the complementary protein and measure protein interactions on the nanoparticle. The protein-coated nanoparticles we used consisted of a magnetic iron core with silver coated on the surface, synthesized using a modified method from [32].

A characterization of the FeAgNP was undertaken using zeta-potential measurements (*n* = 10) to assess surface changes associated with each sample. Values for uncoated NP-C samples (average = 18.04 mV, Std Dev = 3.52), cysteamine-coated NP-CYS (average = 16.28 mV, St Dev = 3.12), and BSA-coated NP-BSA (average = 6.51 mV, St Dev = 1.49) are reported here. The values for other proteins are not reported because the amount of protein needed was cost-prohibitive.

**Figure 3:**
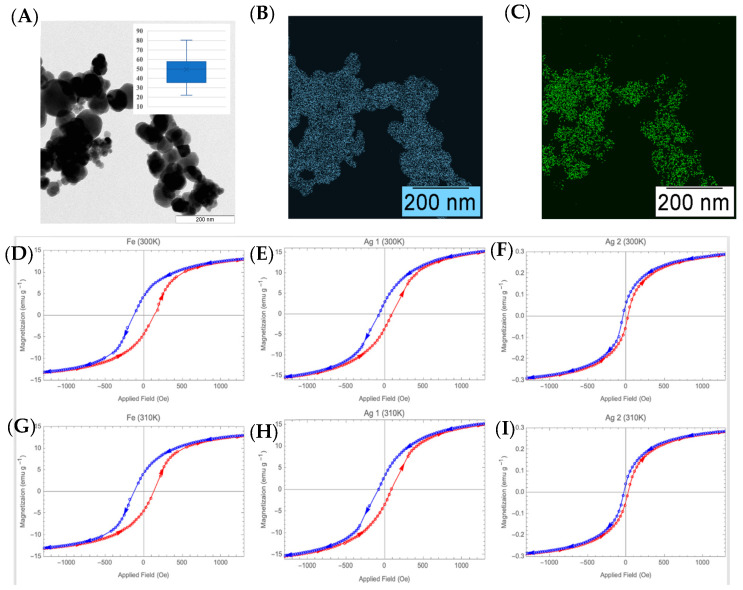
FeAgNP characterization. (**A**) Transmission electron micrograph of FeAgNP, inset: box and whisker plot of nanoparticle size and distribution. (**B**) TEM-EELS map of elemental iron (recolored). (**C**) TEM-EELS map of elemental silver. (**D**–**I**) M vs. H plots of each sample, showing clear hysteresis. The open circles are data points. The solid lines are 3-point moving average fits. The arrows show the direction of the field scan. The colors are redundant, doubly indicating an increasing field (red) or decreasing field (blue). A total of three samples were measured: uncoated iron NPs labeled “Fe” controls (**D**,**G**), and two Ag-coated samples, labeled “Ag1” (**E**,**H**) and “Ag2” (**F**,**H**), at temperatures of 300 K (room temperature) and 310 K (human body temperature).

In addition, Figure 3A displays the results of a TEM image and elemental analysis using EELS. The FeAgNP size range was approximately 50 nm (Figure 3A, inset), and the elemental makeup of the nanoparticles contains both iron (Figure 3B) and silver (Figure 3C). Magnetic characterization was conducted at temperatures of 300 K (room temperature) and 310 K (human body temperature), and hysteresis curves of the magnetic susceptibility were recorded for iron nanoparticles labeled Fe, control in Figure 3D,G; iron nanoparticles with label Ag1, 0.5:1 silver-to-iron ratio (*w*/*w*) in Figure 3E,H; and label Ag2, 1:1 silver-to-iron ratio (*w*/*w*) in Figure 3F,I. The normalized M vs. H curves are shown in Figure 3D–I. From each curve, we extract the hysteresis width. The increasing and decreasing curves do not intersect symmetrically about the origin. We thus report the hysteresis width as an average of the two intersection points (multiplied by 2). It should be noted, the saturation moment of each sample. Fe and Ag1 are similar, with saturation near ~15 emu g^−1^, while that of Ag2 is significantly smaller, ~0.3 emu g^−1^. The sample labeled “Ag2” is believed to have a thicker silver coating than that labeled “Ag1,” thereby suggesting a clear trend between hysteresis width and silver thickness. The presence of hysteresis is perhaps surprising, as it is contrary to M vs. H curves reported by L. Maldonado-Camargo et al., 2017, which show no hysteresis for Fe-Oxide NPs with diameters up to 14 nm [39]. However, it could be related to the larger size of FeAgNP (~50 nm). Using a similar chemical labeling strategy to the coating of the IDE sensors, the nanoparticles were also coated.

**Figure 4:**
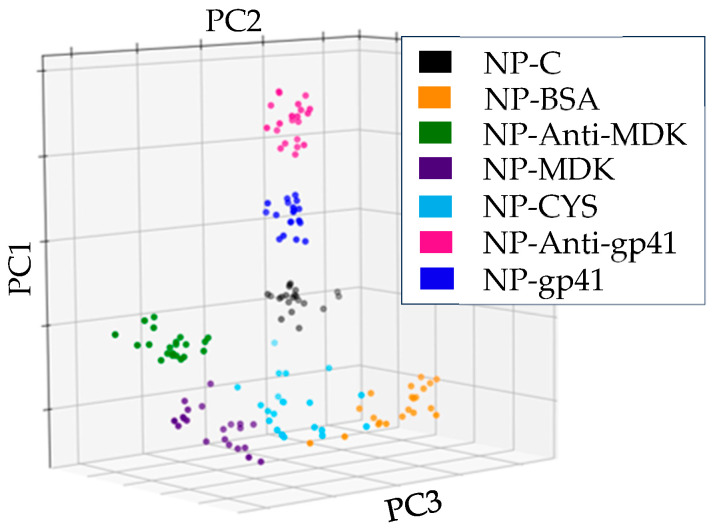
Three-dimensional PCA plots of nanoparticles coated with 5 different proteins compared to uncoated (NP-C) and cysteamine-coated (NP-CYS), generated with impedance data. Results show data clusters based on the identity of the protein coated on the surface (variance ratios: PC1: 0.8513, PC2: 0.1263, PC3: 0.0177) MANOVA *p*-values < 2.00 × 10^−116^, see Supplemental Materials for more detail.

**Figure 5:**
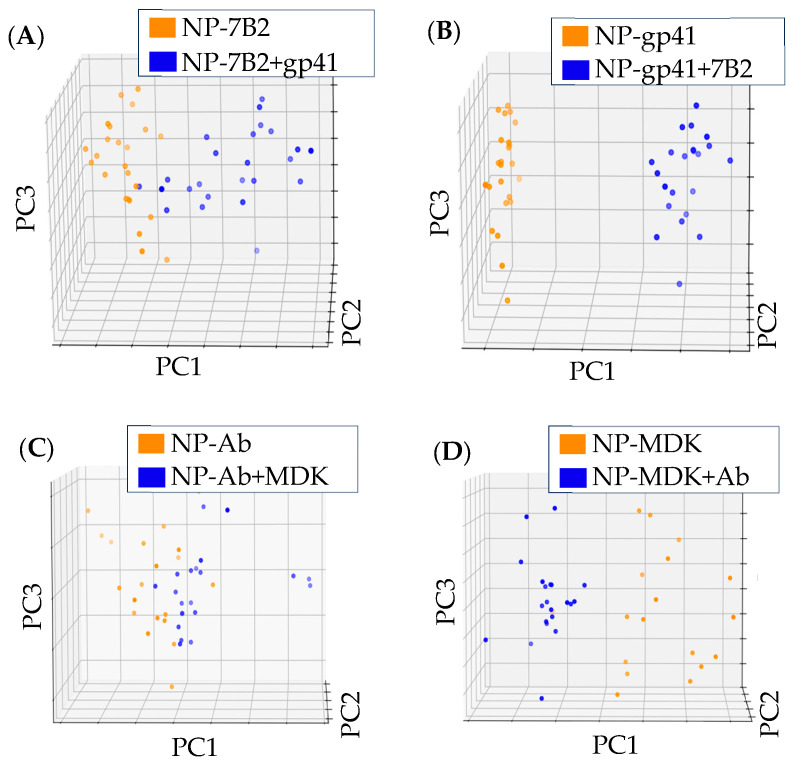
Three-dimensional PCA plots derived from impedance data of nanoparticles coated with an antibody or antigen interacting with their complement. (**A**) Nanoparticles coated with 7B2 antibody before (NP-7B2) and after interaction with gp41 (NP-7B2+gp41), (variance ratios: PC1: 0.9784, PC2: 0.0163, PC3: 0.0038). (**B**) Nanoparticles coated with gp41 before (NP-gp41) and after interaction with 7B2 (NP-gp41+7B2), (variance ratios: PC1: 0.8667, PC2: 0.1061, PC3: 0.0189). (**C**) Nanoparticles coated with MDK antibody before (NP-Ab) and after interaction with MDK (NP-Ab+MDK), (variance ratios: PC1: 0.6823, PC2: 0.2786, PC3: 0.0329). (**D**) Nanoparticles coated with MDK before (NP-MDK) and after interaction with MDK antibody (NP-MDK+Ab), (variance ratios: PC1: 0.9108, PC2: 0.0660, PC3: 0.0203). MANOVA *p*-values < 8.38 × 10^−4^ for all plots; see supplemental data for more detail.

**Figure 6:**
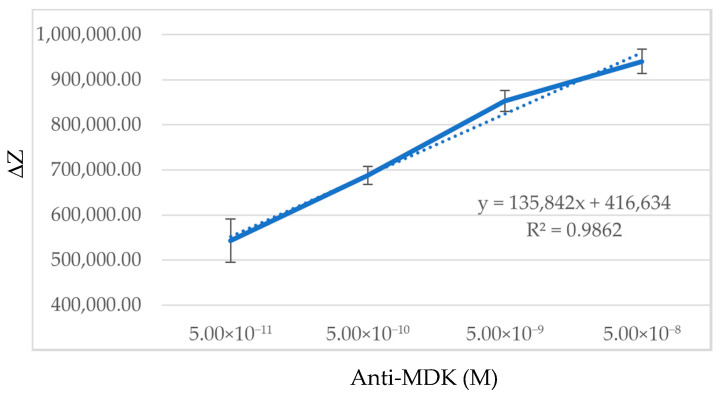
Calibration curve for midkine antibody bound to midkine-labeled nanoparticles (NP-MDK) molarity vs. ΔZ.

Briefly, the FeAgNPs were coated by first reacting CYS with the particle surface while simultaneously reacting the EDC with the protein to be crosslinked after an initial reaction time. After, the coated nanoparticle samples were placed over bare IDE sensors to record the impedance values. When viewing Nyquist and Bode plots, the protein-related changes were less pronounced than when compared to protein-coated IDE sensors. However, by using protein-coated FeAgNP instead of protein-coated sensors, we gained the advantage of using a single IDE many times by just rinsing with DI water 3x between different samples. This, combined with our MUX-8 multiplexor, allowed us to analyze many more individual samples and create much larger datasets than traditional IDE-EIS experiments for validation. Larger datasets helped us to overcome the challenges of sensor-to-sensor variability inherent to the fabrication process, described earlier, by using experiments with larger amounts of samples. To analyze these larger datasets, we came up with an unsupervised data analysis strategy using PCA to help us determine if impedance-related changes could be used to detect interaction. For algorithmic analysis, all data was processed identically without further modification using three steps: (1) Impedance data from Nyquist plots were collated into a single column and scaled between −1 and 1; this allowed data from different sensors to be compared despite sensor-to-sensor variability; (2) Scaled data was transformed into principal components using Scikit principal component analysis [34]; and (3) The first three principal components were plotted on a three-dimensional graph using Matplotlib [35]. The workflow is outlined in Supplemental Figure S1, and the results of this workflow can be visualized in Figure 4, where we have plotted the results from impedance analysis of 7 different classes of FeAgNP, including uncoated (NP-C), cysteamine-coated (NP-CYS), BSA-coated (NP-BSA), anti-MDK-coated (NP-anti-MDK), MDK-coated (NP-MDK), anti-gp41/7B2 (NP-anti-gp41), and gp41-coated (NP-gp41). In Figure 4, each plotted data point represents an individual coated FeAgNP sample, where samples with the same protein coated on the surface cluster together, indicating that the identity of the protein coated on the surface can be inferred by its three-dimensional location on the plot. In Figure 5, we analyzed the ability of the same data analysis strategy to detect protein interaction. Figure 5A compares the FeAgNP coated with mAb 7B2 anti-gp41 (NP-7B2) to anti-gp41 after capture of gp41 peptide (NP-7B2-gp41). Figure 5B shows the opposite orientation where FeAgNPs are coated with gp41 peptide (NP-gp41) and used to capture the Ab (NP-gp41-7B2). Figure 5C,D show the same strategy instead of using anti-MDK and MDK in both orientations. Altogether, Figure 5 suggests that protein-coated FeAgNPs capture complementary proteins, as PCA data clustering shows consistent and distinct impedance-related changes before and after protein capture. The percent coefficient of variation (%CV) was used to assess the reproducibility of both technical replicates and measurements between sensors on scaled data (0 to 1) from NP-MDK samples. A total of 12 impedance curves with 8 different frequencies (0 and 1 omitted) were assessed. This included 4 separate sensors, each with 3 technical replicates. For technical replicates, a %CV was generated at every frequency for each technical replicate and then averaged; the averages were then compared, showing less than 1% variation (%CV = 0.612). For assessing separate sensors, an average %CV was generated at each frequency. The averages were then compared (%CV = 0.857). Data included in Supplementary Materials. In addition, a calibration curve is reported in Figure 6, which plots the molarity (M) vs. unscaled ΔZ at 1 Hz versus different molarities of anti-MDK at 50 pM, 500 pM, 5 nM, and 50 nM attached to NP-MDK.

### 3.4. DNA Coated FeAgNP

**Figure 7:**
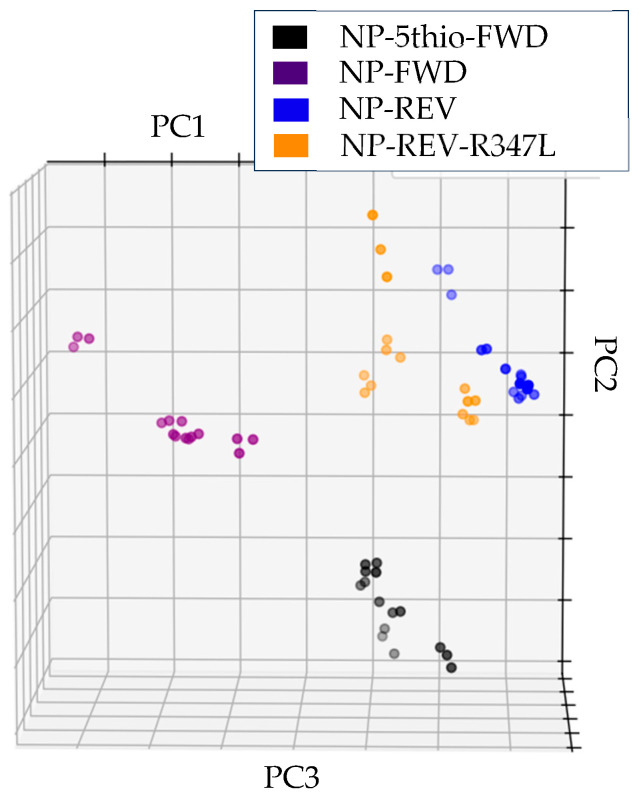
Three-dimensional PCA plot derived from impedance data of nanoparticles coated with DNA (NP-5thio-FWD), after being exposed to negative control DNA (NP-FWD) or positive control complementary DNA (NP-REV) or complementary DNA with a single point mutation (NP-REV-R347L), (variance ratios: PC1: 0.8576, PC2: 0.1254, PC3: 0.0131). MANOVA evaluation of clusters reports *p*-values < 4.92 × 10^−29^ for all metrics; see Supplementary Materials for more details.

We next wanted to test if the same strategy could be used with DNA. To do so, we used a modified labeling method from Wang et al., 2017 [16]. This strategy coated the FeAgNP with a 5-prime thiolated ssDNA sequence (NP-5-thio-FWD) from the Germicidine synthase gene (gcs) from Streptomyces sp. NRRL S-1022. The method was shown to be highly sensitive previously, detecting very low counts of complementary DNA in the previous study. We decided to test if the NP-5-thio-FWD-coated FeAgNP could be used to detect differences between complementary ssDNA (NP-REV) and complementary ssDNA with a single point mutation (NP-REV-R347L). Figure 7 displays the results of this experiment. Compared to the negative control (NP-FWD), the NP-REV and NP-REV-R347L groups are very close yet distinct, suggesting the system not only works for detecting ssDNA interaction, but also can distinguish between 100% complementary ssDNA and ssDNA with a single point mutation.

### 3.5. Mass Spectrometry Analysis of Protein-Coated IDE and FeAgNP

The labeling of the nanoparticles and protein interaction was validated by comparing the amount of protein in the supernatant to that found attached to the FeAgNP. This was performed by analyzing the molecular features or peptide fragments by MALDI mass spectrometry, compared to protein controls. Proteins were analyzed by spotting protein controls or FeAgNP directly onto the MALDI plate. The sensors that were directly coated with protein could also be loaded into the MALDI and analyzed directly, simply by adding matrix over the sensor. A mass list correlated to each protein was constructed as a control to identify the presence or absence of the protein. This mass list was then used to verify the presence or absence of protein on the FeAgNP or IDE sensor directly. Table 1 contains the mass list (*m*/*z*) used to correlate and validate each protein identity. Bruker peptide calibration standard II was used as an internal standard in every sample tested to ensure reliable mass accuracy. 

## 4. Discussion

The transition of biosensor detection technology from non-digital, low-throughput to a digital-based, real-time multicomponent analysis will have a significant impact on several domains. IDE-EIS sensor arrays could be made low-cost and high-density, which would allow for widespread adoption. The ability of IDE-EIS to be used with antibody interaction, protein–protein interaction, and DNA interaction means that it can be broadly applicable to many different biomarkers of multiple biomolecular classes. Additionally, we believe that with development, our FeAgNP IDE-EIS can act as a blueprint for this type of analysis using iron, gold, and silver nanoparticles in general. Other advantages include the potential for IDE sensors to be utilized in perpetuity, integrating with computation resources, and AI-based interpretation. The evidence supporting this idea is based on the current reliability of the sensors, which allow for hundreds of individual measurements with limited deterioration. After ~120 uses, the change in background impedance only increased by <18% at 1000 Hz when analyzing PBS 7.5; however, it does appear that buildup is more dramatic at lower frequencies, as at 10 Hz, buildup was ~61%. We will experiment with more rigorous washing regimes in the future to optimize sensor longevity. Future experimentation will be conducted to better understand the physical parameters related to the changes in the signal. EIS circuit modeling quantitatively reveals interface kinetics, mass transport, surface heterogeneity, film integrity, and reaction rates, turning impedance spectra into actionable sensor metrics like binding constants, corrosion rates, and diffusion coefficients. This will be necessary to better understand the fundamental properties affecting sensor responses. Incorporation of microfluidics for sample handling and magnetic separation will be undertaken as the next steps. The future goals will focus on capturing the biomarkers from raw biofluids, as this is a shortcoming of the present study, where we only used purified proteins and DNA in buffers. In future directions, we will focus on analyzing saliva using IDE-EIS in a similar study to our previous work using microfluidics for real-time biomarker analysis with mass spectrometry [7,40].

## Figures and Tables

**Table 1:** Ionization of small molecules using MALDI.

Identity	Mass List (*m*/*z*)
MDK	263.44, 1297.52, 1319.51, 1386.64, 1620.88, 1642.91, 2094.43
Anti-MDK	199.04, 445.31, 505.99, 2045.92, 2059.76, 2080.03, 1312.87, 1781.73
BSA	457.75, 461.73,473.69, 487.65, 494.63, 593.32, 637.32
mAb-7b2	542.37, 558.25, 560.31, 574.19, 654.84, 877.53, 1367.64, 1639.40
gp41	565.278, 591.27, 623.228, 1191.99, 1470.86

## Data Availability

The raw data supporting the conclusions of this article will be made available by the authors on request.

## Supplementary Materials

The following supporting information can be downloaded at: https://www.mdpi.com/article/10.3390/bioengineering12121334/s1, Figure S1: Data Processing Pipeline.

## Abbreviations

The following abbreviations are used in this manuscript:

IDE	Interdigitated electrode
MDK	Midkine
EIS	Electrochemical impedance spectroscopy
NP	Nanoparticles
PCA	Principal component analysis
HIV	Human immunodeficiency virus
MEMS	Micro-electromechanical systems
FeAgNP	Iron-cored silver-coated nanoparticles
ssDNA	Single-stranded DNA
PVD	Physical vapor deposition
PDMS	Polydimethylsiloxane
EDC	1-ethyl-3-(3-dimethylaminopropyl)carbodiimide
PBS	Phosphate-buffered saline
mAb	Monoclonal Antibody
ssDNA	Single-stranded DNA
DI	Deionized water
VSM	Vibrating sample magnetometer
PPMS	Physical Property Measurement System
EELS	Electron Energy Loss Spectroscopy
MALDI	Matrix-assisted laser desorption ionization mass spectrometry
DHB	2,5-Dihydroxybenzoic acid
IDE-EIS	Interdigitated electrodes with electrochemical impedance spectroscopy
