# Supplementary material for: Multi-Component Analysis of Protein- and DNA-Coated Magnetic Nanoparticles Using Electrochemical Impedance Spectroscopy with Interdigitated Electrode Sensors
Source: Bioengineering (Basel). 2025 Dec 7;12(12):1334. doi: 10.3390/bioengineering12121334 (PMC12729830; doi:10.3390/bioengineering12121334)
Supplement: Supplementary file 1 [file bioengineering-12-01334-s001.zip › bioengineering-3972959-supplementary.pdf]

bioengineering

MDPI

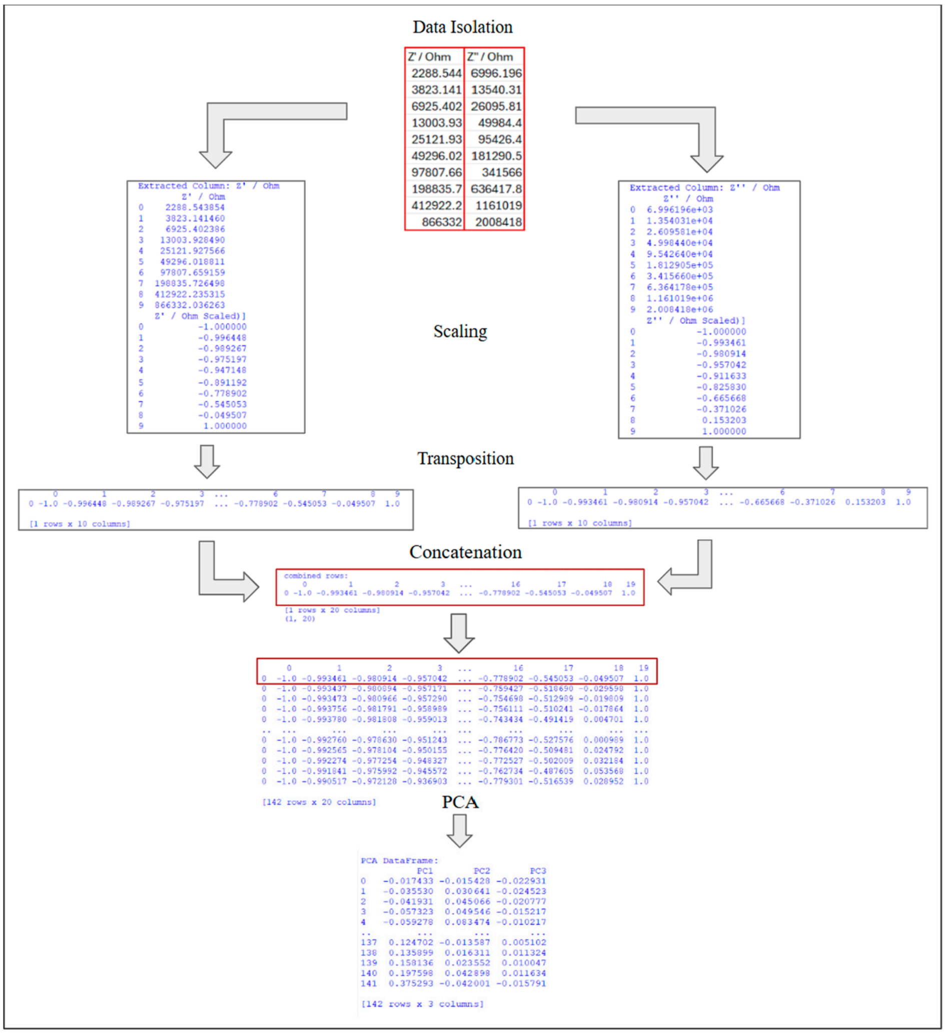

Supplemental Figure S1. Displays the programing process applied to the impedance data. The program started by isolating the impedance data collected on one sensor. Data was scaled, between -1 and 1, and transposed from columns to rows. Using concatenation, one row was added to the end of the other to create a combined row. The combined row was implemented into a larger data frame which contained data from every sensor. PCA application identified the three columns containing the largest variances between the rows, which became the data used for the PCA data frame. Principle components (PC) 1, 2, and 3 became the coordinate points for respective rows that are plotted in the PCA figures.
